# Chemical Food Safety in Europe Under the Spotlight: Principles, Regulatory Framework and Roadmap for Future Directions

**DOI:** 10.3390/foods14091628

**Published:** 2025-05-05

**Authors:** Teresa D’Amore, Slim Smaoui, Theodoros Varzakas

**Affiliations:** 1 Laboratory of Preclinical and Translational Research, IRCCS CROB, Centro di Riferimento Oncologico della Basilicata, 85028 Rionero in Vulture, Italy; 2 Department of Pharmacy, University of Naples Federico II, 80131 Napoli, Italy; 3 Laboratory of Microbial, Enzymatic Biotechnology, and Biomolecules (LBMEB), Center of Biotechnology of Sfax, University of Sfax-Tunisia, Sfax 3029, Tunisia; slim.smaoui@cbs.rnrt.tn; 4 Department of Food Science and Technology, University of the Peloponnese, Antikalamos, 24100 Kalamata, Greece

**Keywords:** chemical food safety, European regulatory framework, chemicals in food, monitoring plans of chemicals, emerging contaminants, food additives and contaminants, pesticides, veterinary drugs, toxins, risk assessment

## Abstract

Chemical food safety is a fundamental pillar of public health, regulatory governance, and economic stability, with far-reaching implications for human, animal, and environmental well-being. In the matter of chemicals in the food chain, the European Union (EU) has established one of the most sophisticated and robust regulatory frameworks to ensure food safety and balance consumer protection with scientific advancements and industry needs. This review provides a holistic analysis of the EU chemical food safety scenario, examining its regulatory framework, key risk assessment methodologies, and the roles of critical institutions involved in monitoring, enforcement, and policymaking. The new and evolving challenges of chemical food safety, including transparency, cumulative risk assessment, and emerging contaminants, were discussed. Special attention is given to major classes of chemical substances in food, their regulatory oversight, and the scientific principles guiding their assessment, as well as to the role of key actors, including regulatory agencies, official laboratories, and competent authorities. This work offers an updated and integrated analysis of chemical food safety in the EU, uniquely combining regulatory, scientific, and enforcement perspectives and providing a structured roadmap for future directions.

## 1. Introduction

Chemical food safety is a matter of fundamental importance within the global regulatory framework, a major challenge in food quality, and has direct implications not only for legislation but also for human, animal, and environmental health, as well as for society and the global economy [1,2].

The European Union (EU) has developed one of the most sophisticated and comprehensive systems for chemical food safety, ensuring the protection of public health and consumer trust while maintaining the integrity of its food supply chain. In particular, the development of a complex legislative framework comprising primary and secondary legislation, along with lower-tier regulatory acts, has the primary objective of protecting consumer health. This framework is built upon key principles, such as precautionary, proportionality, the “no data, no market”, and prevention principles. The precaution principle allows for protective measures in cases of scientific uncertainty, the prevention principle focuses on proactive hazard mitigation, and the proportionality principle requires that risk management measures are commensurate with the identified risks. These principles are interrelated with the “farm-to-fork” approach, addressing potential hazards at every stage of the food production process, from primary production to consumption. The EU commitment to chemical food safety is further reflected in its harmonized legal framework, which includes a combination of horizontal rules—general regulatory provisions that apply across all food categories—and vertical rules, which focus on specific substances or product groups. Together, these regulatory layers establish a cooperative system that governs food safety standards across the Member States (MSs) [3,4,5,6].

The EU governance structure for food safety involves a collaborative interplay among its principal institutions. As is well known, the EU has seven principal decision-making bodies. Among these, the European Commission (EC) is responsible for drafting legislative proposals and overseeing implementation; the European Parliament (EP) scrutinizes, amends, and assures transparency and consumer protection in the legislative process. The Council adopts legislation in consultation with MSs. At the core of this governance framework is the European Food Safety Authority (EFSA), a scientific agency tasked with conducting rigorous risk assessment on chemical substances present in food and feed [7]. The EFSA work spans a broad spectrum of substances, including chemical contaminants (e.g., heavy metals, mycotoxins, dioxins), food additives (e.g., preservatives, sweeteners), environmental pollutants (e.g., per- and polyfluoroalkyl substances—PFASs), veterinary medicinal residues, and substances migrating from food contact materials (FCMs). These scientific evaluations form the basis for risk management decisions taken by EU policymakers and confirm that any authorized substances meet stringent safety criteria. The EFSA collaborates with national food safety agencies and research institutions (the so-called “EFSA Focal Points”) to provide scientifically sound reports and opinions, ensuring that a proper and transparent risk assessment remains at the forefront of policy decisions [8].

The legal instruments available for chemical food safety include regulations, which are binding legislative acts that apply directly in all MSs without the need for national implementation, and directives, which typically set out objectives that MSs must achieve and require transposition into national law through implementing measures by each MS. Additionally, the EU employs a range of lower-tier legislative acts, such as decisions (legally binding for the entities to whom they are addressed and are often used to establish specific measures, such as those taken under crisis management circumstances), recommendations (not legally binding, provide guidance on best practices and policy direction), reports and opinions (inform policy decisions, providing evidence-based guidance on emerging risks and potential regulatory adjustments), which can play a crucial role in identifying data gaps, guiding research needs, and shaping monitoring requirements in the field of chemical food safety [9]. Table 1 provides an overview of these instruments, their characteristics, and examples relevant to chemical food safety.

This structured framework is anchored in Regulation (EC) No. 178/2002, the General Food Law, which establishes the foundational principles for food safety and defines the roles and responsibilities of key actors [7,10,11].

**Table 1:** Legal instrument of chemical food safety in Europe.

Legal Instrument	Description	Binding Nature	Examples	Ref
Regulation	Legislative acts that apply directly in all Member States without national implementation.	Legally binding in all Member States	Regulation (EC) No. 178/2002 (General Food Law) Regulation (EC) No. 1333/2008 (Food Additives)	[7,12]
Directive	Set objectives that Member States must achieve, but each MS chooses how to implement them.	Legally binding, but requires transposition into national law	Directive 2009/128/EC (Pesticide Sustainable Use)	[13]
Decision	Legally binding acts applicable to specific MSs, businesses, or individuals. Often used in crisis management.	Legally binding for the addressed parties	Decision 2002/657/EC (Performance of analytical methods and the interpretation of results)	[14]
Recommendation	Non-binding guidance to encourage best practices and policy direction.	Not legally binding	Commission Recommendation (EU) 2017/84 (Mineral Oil Hydrocarbons in Food) Commission Recommendation (EU) No. 2018/464 (Monitoring of metals and iodine in seaweed, halophytes and products based on seaweed)	[15,16]
Opinion	A formal non-binding instrument used by EU institutions to express views or provide guidance without imposing obligations.	Not legally binding	Opinion of the European Economic and Social Committee on ‘Towards a Fair Food Supply Chain’ (Exploratory opinion) EESC 2021/02472	[17]
Report	Scientific assessments or policy evaluations that inform decision-making.	Not legally binding	Report From the EC to the EP and the Council on food and food ingredients treated with ionizing radiation for the years 2020–2021 COM/2023/676	[18]
Others	Minutes, communication, staff working documents, proposal for a regulation, question.	Not legally binding	Several types of acts	

Despite the robustness of the EU chemical food safety framework, challenges persist. Globalization has significantly increased the complexity of supply chains, introducing new risks such as food fraud, cross-border contamination, and the presence of emerging pollutants linked to industrial activities and climate change [19]. For example, persistent organic pollutants (POPs) such as dioxins, polychlorinated biphenyls (PCBs), and PFASs continue to pose long-term health concerns due to their high persistence and bioaccumulative properties, while newer contaminants, such as microplastics and engineered nanomaterials, present unprecedented challenges that necessitate updated analytical and regulatory strategies, along with enhanced risk assessment methodologies [20,21,22,23,24].

In this complex and continuously evolving scenario, this review aims to provide a comprehensive analysis of chemical food safety within the EU, examining its regulatory framework, risk assessment methodologies, key actors and tools involved in monitoring and enforcement, as well as emerging challenges. It focuses on the major classes of chemicals, providing detailed insights into their regulation and assessment processes. For the first time in the literature, this work harmoniously synthesizes these aspects, offering a roadmap for strengthening the EU chemical food safety framework in an increasingly complex global context. Through this lens, it contributes to ongoing efforts to promote effective risk communication and public awareness while supporting the integration of sustainability objectives into future food policy and regulation.

## 2. Regulatory Framework, Chemical Classes, and Regulations

### 2.1. General Food Law

The foundation of EU Chemical food safety is Regulation (EC) No. 178/2002, commonly referred to as the General Food Law (GFL). Its primary aim is to assure a high level of protection for human health and consumer interests while facilitating the free movement of food within the internal market. The regulation applies to all stages of production, processing, and distribution of food and feed, excluding private domestic activities. It introduces key principles, responsibilities, and procedures to support decision-making on food safety matters and establishes the EFSA as the scientific body responsible for risk assessment. In particular, article 23 details the main twelve tasks of the “Authority”, including providing scientific and technical support to the EC; searching for, collecting, collating, analyzing, and summarizing scientific and technical data, identifying and characterizing emerging risks. EFSA plays a pivotal role under Regulation No. 178/2002 by providing independent scientific advice on food safety issues. Its assessments guide policymakers in setting legal levels of chemicals in foodstuff, such as maximum residue levels (MRLs) for intentionally added substances (e.g., pesticides, veterinary drugs) or maximum levels (MLs) for contaminants (e.g., mycotoxins) [7,18,25,26,27].

Apart from the general principles described above, the GFL introduced several core principles. In article 6, the concept of “Risk analysis” was introduced as the process consisting of three interconnected components: risk assessment (scientific evaluation of hazards and their exposure), risk management (selection of control measures based on assessment outcomes), and risk communication (transparent exchange of information among stakeholders) [28]. The latter has a separate dedicated section since it is essential for ensuring transparency, trust, and understanding among all stakeholders involved in food safety. It is described as the interactive exchange of information and opinions throughout the entire risk analysis process regarding hazards, risks, risk-related factors, and perceptions [29]. This exchange involves risk assessors, risk managers, consumers, food and feed businesses, academics, and other interested parties. Risk communication aims to increase awareness and understanding of specific food safety issues, clarify and disseminate risk management decisions and build public trust in the risk analysis process. Furthermore, it aims to involve all relevant stakeholders appropriately and transparently while providing consumers with information about risk prevention strategies. According to the GFL, risk communication must be consistent with the respective roles of risk assessors and managers. It should ensure that accurate, relevant, and timely information is exchanged interactively with all interested parties based on principles of transparency and inclusivity [30]. 

Transparency has a separate section, and its implementation gave birth to a recent law, Regulation (EU) No. 2019/1381, known as Transparency Regulation (TR) [31]. It has its roots in the glyphosate case and the following EU Citizens’ Initiative and aims to increase transparency, independence, and communication in the EU food safety risk assessment process [32]. The main elements of the TR include ensuring that all studies submitted by industry, excluding commercially sensitive information, are accessible to the public. EFSA must be notified of all commissioned studies, with the responsibility lying with both the industry and the performing laboratories, to prevent the withholding of unfavorable studies and promote more transparency for the public. In cases of strong discrepancies among outcomes, the EFSA can commission additional studies for verification purposes and will perform fact-finding missions to verify the compliance of laboratories and studies with applicable standards. While acknowledging that the TR is not a “panacea” for solving all controversies in regulatory risk assessment, it is a significant step forward toward a better common understanding of regulatory work in the food safety area [29].

Additionally, the GFL mandates traceability, requiring food business operators to track products through all stages of production and distribution to support accountability and facilitate recalls if necessary. Consumer protection is also central, with food law aiming to prevent fraudulent practices, adulteration, and misleading information.

The regulation in article 50 also establishes procedures for managing food safety incidents, including the Rapid Alert System for Food and Feed (RASFF), a tool enabling prompt communication among MSs about risks detected in food and feed [33].

Since its adoption in 2002, the GFL has been amended multiple times to address emerging challenges. Apart from TR, other amendments have refined traceability requirements and expanded the EFSA role in assessing novel hazards like nanomaterials. With other fundamental horizontal rules, such as Official Controls Regulation (EU) No. 2017/625, chemical food safety risks are managed efficiently across MSs [34].

### 2.2. Chemicals in Food

**Figure 1:**
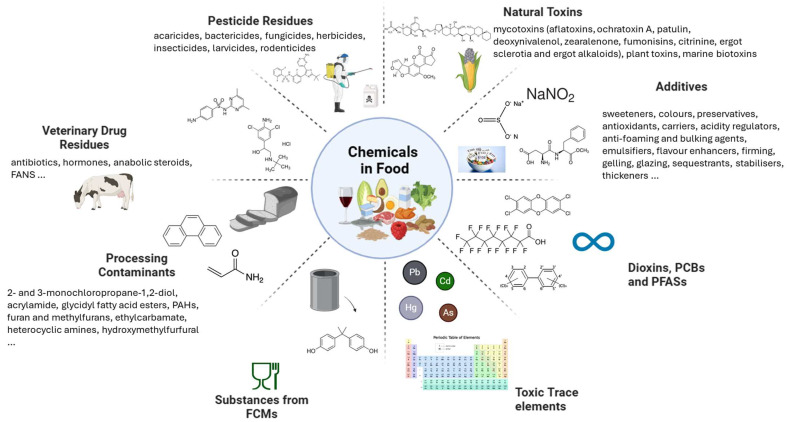
EU-regulated chemicals in food.

On the contrary, vertical regulations target specific chemical classes. An overview of the principal classes of harmful chemicals regulated in the EU is presented in Figure 1.

Chemicals in food and feed can be classified into two broad categories: intentionally added substances and contaminants. Intentionally added substances include compounds deliberately incorporated into food for technological or functional purposes [35]. These include food additives, such as preservatives, emulsifiers, sweeteners, and colorants, which are regulated under Regulation (EC) No. 1333/2008 [11]. These chemicals are strictly monitored since concerns have emerged regarding excessive exposure to food additives, with studies indicating that daily intakes may exceed acceptable levels in certain populations [8,36]. The EFSA, following the EC mandate, periodically reassesses food additives based on new scientific data, particularly for substances with potential health concerns. As an example, the EFSA re-evaluation of glutamates in 2017 found that the exposure to glutamic acid and glutamates exceeded not only the proposed Acceptable Daily Intake (ADI) but also doses associated with adverse effects in humans for some population groups [37]. Similarly, sulfites, benzoates, and artificial colorants have been linked to potential neurological and metabolic health effects, highlighting the need for continuous monitoring and regulatory adjustments [38,39]. In a recent study on the analysis of EU RASFF notifications from 2000 to 2022 for food additives and flavorings, many of them were marked as a significant source of food safety concerns, with sulfites accounting for 40.6% of all notifications between 2000 and 2022. Among the top five hazards reported, benzoic acid, sunset yellow, tartrazine, and erythrosine were frequently flagged due to excessive concentrations, often in soft drinks, apricots, and processed seafood [33,40].

Similarly, pesticide residues result from the application of plant protection products (PPPs) and must comply with MRLs set under Regulation (EC) No. 396/2005 [41]. Moreover, article 32 of this regulation also established that an annual report, which examines pesticide residue levels in the foods on the EU market, is provided by the EFSA. This report is drawn up as part of the EU-Coordinated Control Program (EUCP) that systematically monitors pesticide residues in food. Food products commonly consumed by EU citizens are randomly sampled to provide a statistically representative overview of pesticide residue levels in these products. The findings from the 2022 EU report on pesticide residues in food provide valuable insights into the effectiveness of EU regulatory measures [42]. The report highlights that 96.3% of the samples analyzed complied with legal limits, with a slight decrease in MRL exceedances compared to previous years. However, non-compliance rates in imported products from third countries were found to be significantly higher than those of domestically produced food, reinforcing the need for stringent import controls and continued monitoring. The latest Commission Implementing Regulation (EU) No. 2024/989 outlines a three-year monitoring cycle (2025–2027), randomly sampling commonly consumed food products to assess consumer exposure and detect compliance with MRLs [43,44]. 

In addition, Regulation (EC) No. 1107/2009 governs the authorization of PPPs, requiring active substances to undergo scientific risk assessment by EFSA and MSs before approval. It also includes provisions for banning hazardous substances, setting protection zones, and restricting pesticide use based on risk assessments [45,46].

Veterinary drug residues arising from the treatment of food-producing animals are regulated under Regulation (EU) No. 37/2010, which establishes acceptable residue levels in animal-derived food products [47,48].

Additionally, chemicals from FCMs have two dedicated regulations: providing the general principles and establishing Specific Migration Limits (SMLs) for individual substances (i.e., the maximum permitted amount, usually in mg/kg food) that may migrate into food or food simulants), and the authorization procedures. A Union List of authorized substances that may be used in the manufacture of plastics was also provided. SMLs are based on toxicological evaluations conducted by the EFSA, considering reference values and exposure scenarios. Manufacturers must conduct migration testing under worst-case conditions to demonstrate compliance, using standardized food simulants and test conditions that reflect the intended use of the material [49,50]. For Bisphenol A, classified as a substance of very high concern (SVHC) due to its endocrine-disrupting properties for humans, in 2024, the EC published Regulation (EU) No. 2024/3190, which prohibited it and other bisphenols and bisphenol derivatives use in all FCMs [51,52,53].

In contrast, contaminants are unintended substances that may enter food through environmental pollution, agricultural activities, or industrial processes. They have the most complex regulations as a consequence of their multifaceted chemical properties. In fact, this class includes inorganic chemicals (toxic trace elements—TTEs, nitrates, perchlorates) and organic chemicals. These latter comprise all mycotoxins, natural toxins, and POPs, such as dioxins and PCBs. In 2023, the EC adopted Regulation (EU) No. 2023/915, which replaced the earlier Regulation (EC) No. 1881/2006 [54]. The previous regulation had undergone nearly fifty amendments due to the continuous addition of new contaminants, food categories, and updated scientific findings. The new regulation consolidates this complex and evolving body of legislation, offering a unified framework for setting and managing MLs in food. To guarantee product safety, these levels are established following the “as low as reasonably achievable” (ALARA) principle, relying on good agricultural and manufacturing practices to minimize contamination [55,56]. Particular attention is given to high-risk food categories, which are more susceptible to certain contaminants and, therefore, require regular testing to monitor that thresholds are not exceeded. Among the most scrutinized contaminants are mycotoxins such as aflatoxins, ochratoxin A, patulin, deoxynivalenol, zearalenone, fumonisins, citrinin, T-2, and HT-2 toxins. These are toxic metabolites produced by fungi, commonly found in cereals, fruits, and derived products, and many are considered carcinogenic or genotoxic by the EFSA [57,58,59,60,61]. Similarly, natural plant alkaloids like tropane, ergot, and pyrrolizidine alkaloids pose significant health concerns, particularly for vulnerable groups such as infants and children [62,63,64,65]. TTEs, such as lead, cadmium, mercury, tin, and arsenic, are also tightly regulated due to their persistence and bioaccumulative properties [66,67]. A challenge for TTEs in food safety is that some of them may assume organic forms in some matrices. As an example, arsenic exists in both organic and inorganic forms. Organic arsenic compounds, such as arsenobetaine found in seafood, are considered to have lower toxicity. In contrast, inorganic arsenic (as a sum of As^III^ and AS^V^) is highly toxic and classified as a Group 1 carcinogen by the International Agency for Research on Cancer (IARC). Regulation (EU) No. 2023/915 sets MLs specifically for inorganic arsenic, especially in rice and rice-based products, due to its prevalence and risk to human health. Speciation is required in this case, as it is necessary to distinguish toxic inorganic arsenic from the non-toxic organic forms [68,69,70,71].

For some process contaminants, *ad hoc* laws were set. This is the case of acrylamide, which forms naturally when foods rich in free asparagine and sugars are subjected to high-temperature cooking methods, such as frying, roasting, and baking. Since its discovery in food in 2002, extensive research has been conducted to develop mitigation strategies. In response, FoodDrinkEurope created the Acrylamide Toolbox to guide the food industry in reducing acrylamide formation. The EC has also issued recommendations for monitoring acrylamide levels in food and assessing industry compliance. The EFSA evaluated the risks of acrylamide in 2015, confirming its carcinogenic potential and raising concerns about dietary exposure. The main sources of acrylamide intake include fried potato products, baked cereals, and coffee [72,73]. However, investigations revealed inconsistent application of mitigation measures across food businesses, ranging from full compliance to no action taken. To aid enforcement, a harmonized guidance document was developed to standardize compliance across the EU. Further measures were introduced under Recommendation (EU) No. 2019/1888, encouraging expanded monitoring of acrylamide in foods not previously covered by regulations but potentially contributing to dietary exposure [74]. Discussions are also ongoing to establish MLs of acrylamide in additional food categories, particularly processed cereal-based foods for infants and young children.

Similarly, *Alternaria* toxins (ATs) have been identified as new high concerning contaminants due to toxicological evidence indicating their potential to damage DNA. Following two EFSA reports, the EC issued Recommendation (EU) No. 2022/553, establishing indicative levels for major ATs in products such as cereals, tomato-based foods, spices, oilseeds, and baby food [75,76,77]. Nevertheless, despite growing scientific evidence highlighting their risks, global regulations and established MRLs for these toxins remain absent [78].

On the contrary, there is a master law for chemicals in feed, Directive 2002/32/EC, which sets up undesirable substances in animal feed materials, compound feed, and complete feed [79,80].

To effectively regulate these chemical classes, the EU has adopted various legislative instruments. Table 2 provides an overview of the main regulations governing different categories of chemicals in food and feed.

### 2.3. Controversies

Although the EU has developed a sophisticated and comprehensive regulatory framework for chemical food safety, over the years several controversies have come out regarding the performance and independence of its key scientific body, as well as accusations of potential conflicts of interest among risk assessors and managers. In particular, criticisms were raised about potential conflicts of interest among EFSA experts, and the transparency of its risk assessment processes [86,87,88]. Reports published by Corporate Europe Observatory between 2012 and 2017 documented that a significant proportion of EFSA panel members had financial ties to industry, raising concerns about the Authority’s independence [87]. In response, the EFSA has subsequently strengthened its conflict-of-interest policies. Additionally, the appointment processes for experts and panel members have become more regulated, with MSs playing a more significant role. However, debates about the effectiveness of these measures persist.

High-profile cases, such as the evaluation of glyphosate carcinogenicity, have further fueled criticism. Divergences between the EFSA assessment and that of the IARC illustrated differences in methodologies, transparency, and regulatory approaches [89,90]. IARC classified glyphosate as a probable human carcinogen based on sufficient evidence in animals, limited evidence in humans, and strong mechanistic data, whereas EFSA concluded that glyphosate is unlikely to pose a carcinogenic risk. This finding was criticized for its reliance on unpublished industry studies, dismissal of epidemiological and animal data, and lack of transparency. The latest glyphosate reauthorization process has exposed deep-rooted tensions within the EU regulatory epistemology, highlighting persistent challenges regarding transparency, independence, and public trust in food governance and demonstrating how scientific, legal, and political contestations can profoundly shape both risk assessment and risk management frameworks [32,91,92].

Similar concerns have surrounded EFSA evaluations of genetically modified foods, gene-edited organisms, endocrine-disrupting properties, and food additives like aspartame [93,94,95,96].

In response to these concerns, particularly in the field of food safety, the EC disposed of public access to data—“science and secrecy do not sit comfortably together”—and improved stakeholder participation, as well as the role of MSs in risk assessment [97]. Additional initiatives, such as those promoted by the Pesticide Action Network (PAN Europe), also contributed to making pesticide risk assessment reports publicly accessible [98].

Together, these efforts, alongside novel policies for the responsible use of chemicals, contributed to a broader re-evaluation of regulatory frameworks, including those governing PPPs and biocides, which lead, as an example, to the introduction of hazard assessment of endocrine disruptors and chemicals toxic to pollinators. Major initiatives such as the Chemicals Strategy for Sustainability, the Regulatory Fitness and Performance Programme (REFIT), and the EU Biodiversity Strategy were launched, accompanied by the publication of several new guidelines promoting more transparent scientific evaluation and risk assessment processes [99,100,101].

## 3. Risk Assessment of Chemicals in Food

Risk assessment (RA) is a rigorous, structured, multidisciplinary, and iterative process that aims to address questions related to exposure to one or more chemical, physical, or biological agents that pose potential risks to human health and the environment. Over time, RA has evolved into a specialized scientific discipline involving the analysis and review of scientific data to estimate the probability of adverse events resulting from exposure to hazardous substances (Risk = Hazard × Exposure) [102].

At the heart of RA are regulatory toxicology and toxicological testing, which serve as fundamental tools to guide experts and regulatory bodies in decision-making across various industrial and regulatory sectors, including chemicals, pharmaceuticals, pesticides, cosmetics, veterinary drugs, novel foods, polymers, special mixtures, recycled materials, and biocides [103].

Scientific and technological advancements, alongside a growing awareness among regulatory authorities, researchers, and the food industry, have driven the need for a deeper understanding of the toxicological profile (“fingerprinting”) of chemical substances. This applies not only to new substances requiring approval before commercialization but also to known substances already present in the environment.

Given the complexity of chemical exposure scenarios, there is an increasing need to develop, optimize, and validate new tools capable of assessing risk across a broad range of substances, ensuring safety while addressing modern toxicological challenges [104,105,106]. The key objectives of RA research and development are as follows:

- Reducing or replacing animal testing by using *in vitro*, *in silico*, and *in chemico* models;
- Identifying, evaluating, and minimizing uncertainties in exposure assessments.
- Filling knowledge gaps, particularly in mechanistic toxicology and exposure modeling;
- Assessing the effects of exposure to chemical mixtures, including multiple chemicals and other stressors.

RA is conducted in four main stages, preceded by a preliminary phase:

(0) Data collection and information gathering.

(1) Hazard identification (“identification of the kind and nature of opposing impacts that an agent with a characteristic ability to provoke an impact on organism, system or population”).

(2) Hazard characterization (“the qualitative and quantitative description of the intrinsic properties of an agent or condition with a potential to lead to opposing effects”).

(3) Exposure assessment.

(4) Risk characterization, which integrates the previous steps to determine the qualitative and quantitative probability of adverse effects under specific exposure conditions, including associated uncertainties [107].

The conclusions of this process serve as the basis for risk management decisions, leading to the implementation of Risk Mitigation Measures, such as use restrictions, maximum exposure limits, and population-specific safety measures [103].

A crucial aspect of RA is the dose–response relationship, which quantifies how an external dose (exposure assessment) translates into an internal biologically active dose (toxicokinetics—TK), ultimately leading to toxic effects (toxicodynamics—TD). The next step is to determine the dose descriptors. The derivation of these values is always performed by experts, institutions, and regulatory agencies that analyze toxicological studies, the most part long-term *in vivo*, but also novel methodologies based on *in vitro*, *ex vivo*, and *in silico* studies stand-alone or integrated [102]. These studies are useful to derive a reference point, the point of departure (PoD), or “the point on a dose–response curve established from experimental data used to derive a safe level”. Among them, the most traditionally used are the NOAEL (no observed adverse effect level) and the LOAEL (low observed adverse effect level), which represent the highest concentration of a substance at which no measurable effect or the lowest measurable effect, respectively, occurs in a given population exposed to that substance. Other approaches have recently been introduce to overcome some limitations of NOAEL, such as the benchmark dose BMD, the minimum dose of a substance that produces a clear, low-level health risk, usually in the range of a 1–10% change in a specific toxic effect such as cancer induction. The BMD methodology, predicted on a mathematical model fitting to the experimental dose–response material, is preferred for both cancer and non-cancer endpoints. Once referred to a carcinogenicity investigation, the BMD approach was calculated as the lower limit of the projected dose at 10% (BMDL_10_) extra tumor occurrence [108]. Descriptive assessments of published BMD data (Table 3) revealed that appraisals resulting from dissimilar dose–response datasets for the same compound could diverge considerably. Moreover, across genotoxicity endpoints, the difference can extend an order of magnitude or more. A comparable level of variability is observed in BMDL values stemming from a similar endpoint but restrained in different organs or tissues. In some instances, BMD estimates also seemed to be predisposed by investigational situations like exposure duration or synchronization of tissue collection. These explanations propose that the effects of a dose–response examination built on genotoxicity could be meaningfully touched by the endpoint selection, specific target organ toxicity, and study restrictions.

These PoDs are used to calculate the health-based guidance values (HBGVs). For chemical substances intentionally added to foods, such as food additives, pesticide residues, and veterinary drugs, the HBGV used is the ADI, while for contaminants (e.g., TTEs), which are not added deliberately to foods, tolerable daily intake (TDI) and the tolerable upper intake level (UL) for nutrients (e.g., vitamins and minerals) are adopted for the general population or specific subgroups. It is an estimate of the amount of a substance in food/feed and beverage that can be ingested daily over a lifetime without appreciable risk to health. The HBGV is usually expressed as milligrams of the substance per kilogram of body weight per day (mg/kg/day; standard human = 60 kg). It is calculated as a ratio of the PoD (NOAEL, LOAEL, BMDL, etc.) and uncertainty factors or assessment factors (UFs). The UFs are dimensionless values, which include the contributions of all uncertainties arising from interspecies differences between humans and animals, as well as interspecies differences within the human population. Other UFs, deriving from uncertainties in the toxicological studies conducted, such as the use of LOAEL instead of NOAEL or the unavailability of long-term studies, can be added by risk assessors based on expert judgment and the weight of evidence available (WoE approach). Typically, the total safety factor is 100 (10 for species differences and 10 for human variability).

Exposure levels are then compared to reference values using risk characterization methods, such as Margin of Safety (MoS), margin of exposure (MoE), and Risk Characterization Ratio (RCR), to draw conclusions on potential health risks [108,116].

The RCR, also known as the hazard quotient, is a ratio used to compare the predicted exposure to a chemical or pollutant with an HBGVS. It is a key step in RA, helping determine if the potential harm from a substance is likely to be significant.

The RCR is typically calculated as follows:

*RCR = Exposure/HBGV* (1)

An RCR of 1 or less (RCR ≤ 1) generally indicates an acceptable level of risk, meaning the exposure is below the benchmark.

An RCR greater than 1 (RCR > 1) suggests that the exposure may exceed the safe level and potentially pose a risk.

Another similar approach developed for non-genotoxic chemicals by Doménech and Martorell is the MoS or probabilistic food safety margin (p-FSM):


*MoS = HBGV/Exposure* (2)

The value obtained varied from 0 to 1, so a rate of ~1 designates an extensive margin. Exposure to this hazard is highly improbable to have health significance, while a margin of ~0 suggests a strong probability of a non-genotoxic opposing effect.

Conversely, to assist in risk management for hazards with genotoxic impacts, the JECFA (Joint FAO/WHO Expert Committee on Food Additives) and the EFSA developed the MoE. This approach does not brand implicit assumptions regarding safe consumption and was extensively employed to evaluate substances that are both carcinogenic and genotoxic [56]. The MoE is computed as the ratio between a PoD and an estimated human exposure level.


*MoE = PoD/Exposure* (3)

Otherwise, some researchers employed the hazard index or risk quotient for genotoxic effects. On the other hand, the excess cancer risk can also be considered when calculating the margin for genotoxic hazards. It can be predictable in terms of the accumulative probability of emerging cancer over a lifetime of total exposure to a possible human carcinogen. The cancer benchmark concentration is measured by partitioning the maximum acceptable risk level (1 × 10^−6^) by the slope factor, multiplying the value achieved by the body weight, and dividing this outcome by the consumption [117].

**Figure 2:**
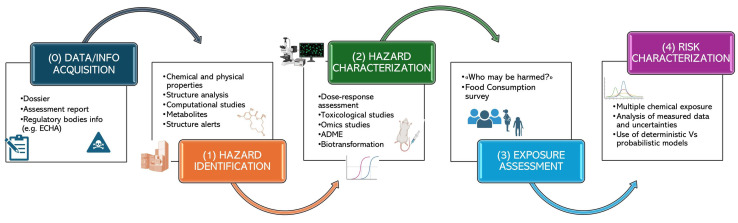
Risk assessment stages of chemicals in food.

While these steps are conceptually distinct, they are inherently interconnected and continuously evolving, guided by the principles of transparency, integration, and scientific progress. RA has thus become a dynamic, inductive-deductive scientific process capable of adapting to new evidence and technological advancements. In Figure 2, a summary of these steps is presented.

Historically, hazard identification and characterization relied heavily on animal studies. While OECD test guidelines and Good Laboratory Practices (GLP) have standardized these methods, animal testing remains resource-intensive, costly, and ethically challenging. Consequently, modern toxicology has shifted toward New Approach Methodologies (NAMs), which include *in vitro*, *in silico*, and alternative testing strategies aimed at reducing reliance on traditional animal models. In support of the use of NAMs, there is the long-established key concept 3Rs (Replacement, Reduction, and Refinement) first introduced in 1959 by British academics William Russel and Rex Burch, together with thousands of global, national, and local initiatives and projects. These principles form the foundation of EU Directive No. 63/2010 to reduce animal testing by promoting alternative, reliable methods for assessing human and environmental risks while maintaining scientific and ethical standards. In this way, NAMs guide the transition toward new human-relevant toxicological strategies, representing the tools of Next Generation Risk Assessment (NGRA) [118,119,120].

A key issue in chemical food safety RA is cumulative exposure to multiple substances, particularly those that share toxicological mechanisms (Mode of Action—MoA), such as endocrine disruptors, neurotoxic compounds, and POPs. Traditional RA methodologies have largely focused on single-substance evaluations, but increasing attention is being given to mixture effects, recognizing that combined exposure to multiple chemicals, even at low levels, may result in adverse health outcomes. This has led to the adoption of cumulative risk assessment (CRA) approaches, particularly for pesticide residues and food additives [20,121].

### Cumulative Risk Assessment of Chemicals in Food

Human exposure occurs to a complex mixture of chemicals through various sources, including diet. CRA represents a paradigm shift in this field, moving beyond the single-substance approach to evaluate the combined health risks associated with simultaneous exposure to multiple chemicals. This approach acknowledges that even substances present at levels considered safe individually might pose a risk when combined, as their effects can add up or interact. The need for CRA is increasingly recognized due to the ubiquitous presence of chemical mixtures in the food supply and the environment, necessitating a more comprehensive understanding of potential health impacts. The sheer number of potential combinations of chemicals in food further underscores the complexity and growing importance of assessing their cumulative impact on human health [106,122].

The significance of CRA in EU chemical food safety lies in its potential to provide a more accurate estimation of risks to the population. By considering the combined effects of chemicals with similar toxicological profiles, CRA can identify potential hazards that might be overlooked by single-substance assessments. For instance, pesticides that individually might not exceed safety limits could collectively pose a risk due to their additive effects on a particular organ system [123].

Given the limitations of single-substance RA, the EFSA has been tasked with developing and implementing methodologies for CRA, particularly for pesticides. A key concept of the EFSA approach to CRA is the Cumulative Assessment Groups (CAGs). CAGs are formed by grouping substances, primarily pesticides, that exhibit similar toxicological effects on specific organs or systems. The underlying assumption is that pesticides causing the same toxic effects can produce a joint, cumulative toxicity, even if they do not share similar MoA. EFSA has established a procedure for defining these CAGs based on the common toxicological effects of pesticides [124,125].

Although the overall process of CRA follows the standard four steps of the RA paradigm, exposure assessment in the context of CRA involves determining the dietary exposure to the chemical mixtures of concern. This is often achieved by utilizing individual food consumption data collected through national food consumption surveys and occurrence data gathered by MSs under their official monitoring programs. The assessment for chemical mixtures relies on the principle of dose addition for substances that exert similar toxicological effects. Dose addition assumes that the combined effect of multiple chemicals acting through the same MoA or on the same target organ is the sum of their individual effects. While dose addition is a common starting point, EFSA also acknowledges the potential for other types of interactions, such as synergism (where the combined effect is greater than the sum of individual effects) and antagonism (where the combined effect is less than the sum of individual effects). RCR involves integrating exposure and hazard information to estimate the likelihood and severity of adverse health effects.

The primary tools used include the hazard index (HI), Relative Potency Factor (RPF), and Toxic Equivalency Factor (TEF) methods, which allow for cumulative risk quantification by normalizing different chemicals to a reference compound. Also, these methodologies enable regulators to estimate total exposure levels from multiple substances and determine whether cumulative exposure exceeds health-based safety thresholds.

The most up-to-date approach for CRA is the tiered approach for calculating exposure to CAGs. The first step in this approach involves identifying a toxicological effect that can plausibly be caused by multiple chemicals. Once such effects are identified, substances causing these effects are included in CAGs and characterized for the specific effects by establishing reference values.

The exposure assessment then follows a two-tiered (or sometimes three) structure. In Tier 1, more generic parameters are used, leading to a conservative assessment. Tier 2 refines the assessment using more realistic, yet still conservative, input parameters. This tiered approach aims to save resources by avoiding further assessment in Tier 2 if Tier 1 indicates no risk. Both tiers utilize probabilistic modeling, a shift from deterministic approaches that rely on single values. Probabilistic assessments use distributions of values, combining consumption data from dietary surveys for all food commodities (converted to Raw Primary Commodities—RPCs) with analyte concentrations retrieved from the EU Multi-Annual Control Programmes over a 3-year monitoring cycle (for pesticides in particular). This generates a distribution of consumer exposures to pesticide residues, representative of all age classes and countries in the Pesticide Residues Intake Model (PRIMo). The EU MACP, although ideally based on random sampling, also includes samples from MSs’ National Control Programmes to increase sample numbers, potentially introducing some bias due to selective sampling. Enforcement samples, which target known issues, are excluded to ensure a representative market overview. Samples exceeding MRLs are included to provide a realistic market picture.

The risk characterization outcome is communicated using the concept of total margin of exposure (MoET). The MoET represents a safety margin for a group of substances, comparing human exposure to levels causing adverse health effects, using substance-specific toxicological data and relative potencies. Both consumers and non-consumers of an RPC are included in both tiers to provide the best exposure estimate. An indicative target MoET of 100 at the 99.9th percentile of the total population is used as a threshold for regulatory consideration, consistent with the safety margin for establishing toxicological reference values. A MoET above 100 suggests a sufficient safety margin, likely negating the need for regulatory action. A MoET below 100 for a certain population percentage does not automatically indicate risk but suggests that risk managers should consider action.

EFSA has conducted several pilot CRAs, primarily focusing on pesticide residues and their potential effects on specific organ systems. One significant area of work was conducted on pesticides affecting the nervous system. In April 2020, EFSA delivered initial reports assessing the cumulative risk of pesticide residues with both acute and chronic effects on the nervous system, utilizing EU monitoring data from 2014 to 2016. Prior to these assessments, EFSA had established CAGs for pesticides known to affect the nervous system. These CAGs were defined for five distinct effects on the nervous system, including inhibition of brain and/or erythrocyte acetylcholinesterase; functional alterations of the motor, sensory, and autonomic divisions; and histological neuropathological changes in neural tissue. The overall conclusion of these pilot assessments was that the consumer risk from dietary cumulative exposure to these pesticides was, with varying degrees of certainty, below the threshold that would trigger regulatory action [125].

Similarly, EFSA assessed the cumulative risk of pesticide residues with chronic effects on the thyroid system. CAGs were established for pesticides affecting the thyroid, specifically for two effects: hypothyroidism and parafollicular cell (C-cell) hypertrophy, hyperplasia, and neoplasia. The conclusion of the thyroid assessment mirrored that of the nervous system assessment, indicating that cumulative exposure to these pesticides did not exceed the regulatory threshold [124].

More recently, in 2022, the EFSA conducted a dietary CRA in a retrospective manner focusing on the potential impact of pesticide residues on craniofacial alterations in women of childbearing age. This assessment considered two types of craniofacial alterations and was performed for 14 EU populations of women in this vulnerable life stage. The findings of this assessment indicated that the MoET resulting from cumulative exposure to pesticide residues was above 100 for both types of craniofacial alterations, suggesting that the risk was below the established regulatory threshold [126].

To facilitate the scientific assessment, various tools (e.g., Rapid Assessment of Contaminant Exposure—RACE, OpenFoodTox …), as well as repositories and platforms (e.g., Zenodo), were developed and commissioned to enable the swift processing of information, support consistent analysis and reporting of results, and facilitate predictive modeling [127,128,129].

## 4. Actors of Chemical Food Safety and Role of Analytical Controls and Monitoring Studies

The enforcement of the regulations described above is supported by a multi-tiered control system, including routine inspections, laboratory testing, and food sampling programs conducted by MSs. This regulatory framework is primarily governed by Regulation (EU) No. 2017/625 (Official Controls Regulation, OCR) and Regulation (EU) No. 2019/627, which provide a harmonized structure for official controls along the entire food chain [34,81].

The OCR establishes a harmonized framework for official controls and other official activities performed to support the application of food and feed laws, animal health and welfare rules, plant health regulations, and rules concerning PPPs. This regulation consolidates and simplifies the legislative framework for official controls by repealing and replacing several previous regulations and directives, including Regulations (EC) No. 854/2004 and (EC) No. 882/2004. Competent authorities within each MS implement coordinated strategies for inspection, sampling, and laboratory analysis to detect chemical contaminants, veterinary drug residues, and other hazardous substances in food and feed. The OCR is a horizontal law that applies to diverse areas, including food and feed hygiene, zoonoses, animal by-products, contaminants, food labeling, genetically modified organisms, and organic production. Competent authorities within MSs are responsible for conducting official controls, including routine and risk-based inspections, laboratory testing and verification activities, imposing enforcement actions for non-compliance, and targeted sampling programs [66,130].

Under this regulation, each MS must designate a single authority to coordinate food safety measures and facilitate communication with other MSs and the EC. To enhance the effectiveness of food safety control, the OCR mandates the development of several multi-annual control plans, ensuring a systematic and structured approach to food safety monitoring.

Regulation (EU) No. 2019/627 provides specific rules for the performance of official controls in food production, particularly in meat and dairy processing establishments. This regulation defines detailed procedures for sampling and laboratory analyses to monitor compliance with microbiological and chemical safety criteria [81].

### Analytical Controls and Monitoring Studies in Chemical Food Safety

In this integrated and holistic system, a critical role is played by analytical studies and monitoring. A robust analytical control system is essential for detecting and quantifying chemical contaminants in food. The presence of harmful substances, often at trace level, necessitates highly sensitive and validated analytical techniques [68,131].

The implementation of analytical controls is guided by several key regulations and decisions, including the following:

- Regulation (EU) No. 333/2007: Establishes criteria for the detection of heavy metals in foodstuffs [132].
- Regulation (EU) No. 2017/625: Provides a framework for food safety controls [34].
- Regulation (EU) No. 2019/627: Defines procedures for official laboratory testing [81].
- Decision 2002/657/EC: Specifies criteria for the validation of analytical methods for veterinary drug residue detection [13].
- Regulation (EU) No. 2021/808: Updates method performance criteria for residue analysis.

The analytical methodologies employed in chemical food safety monitoring must be robust, precise, and reproducible. The performance of these methods is ensured through adherence to reference standards and validation protocols [133].

Accurate detection of contaminants is based on validated analytical methods. Method validation is a fundamental process in chemical analysis, essential for assuring the accuracy and reliability of analytical results. The complexity of a method directly influences the extent and depth of validation required, with more intricate techniques demanding a more rigorous and comprehensive validation process. The ISO/IEC 17025, the international standard for testing and calibration laboratories, establishes general requirements for method validation and laboratory competence [134]. A summary of validation parameters is provided in Table 4.

Reference methods used in regulatory food testing must comply with the criteria outlined in Regulation (EU) No. 2021/808, which updates method performance standards for residue analysis [133]. These methods go through rigorous validation procedures to assure compliance with EU food safety regulations. All methods must be developed in accordance with Good Laboratory Practices (GLP). GLP principles, as defined in Directive 2004/9/EC, along with ISO/IEC 17025, require quality assurance protocols, standardized documentation, and staff training to support the reliability and traceability of analytical results [134].

**Figure 3:**
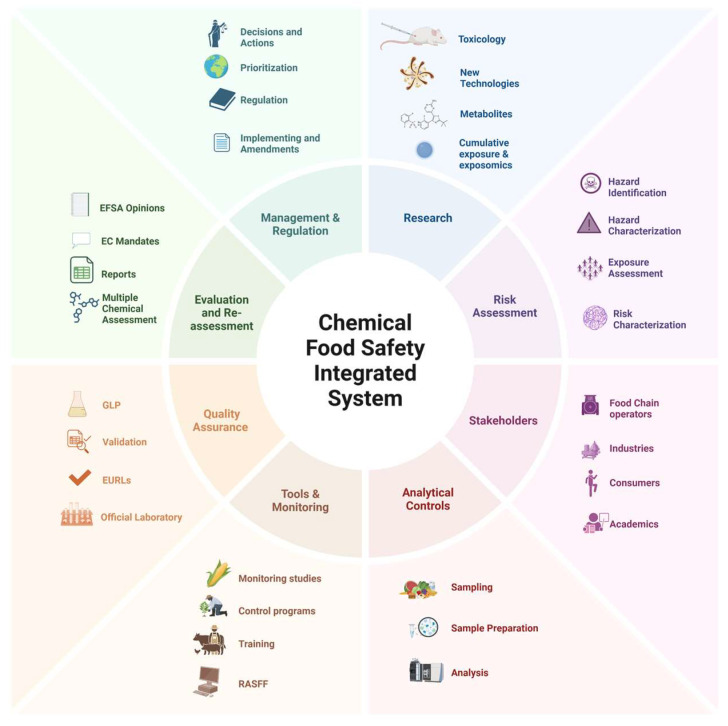
Chemical food safety integrated approach.

Analysis of chemicals in food and feed along the food chain guarantees their correct assessment and management and has a pivotal role in minimizing exposure worldwide [136]. The resulting scenario derived from analytical controls helps support decision-making and control plans. In addition, in this holistic system, analytical controls and monitoring studies are intimately connected with the new developments and research strategies [57]. A summary of the chemical food safety integrated approach is provided in Figure 3.

The supervision of this integrated system is mandated by the Directorate-General for Health and Food Safety (DG SANTE), which is responsible for the monitoring and implementation of EU policies and laws also in the matter of chemical food safety. It also develops documents and guidelines for ensuring the correct development of analytical methods, such as the SANTE 11312/2021 for pesticide residues in food and feed [137].

Finally, to assure uniformity and high analytical standards across the EU, the European Reference Laboratories (EURLs) play a critical role in harmonizing testing methods, providing training, and supporting MS official laboratories in implementing best practices. EURLs carry out inter-laboratory comparisons (ILCs), proficiency tests (PTs), and method standardization to maintain uniformity in chemical food safety testing.

## 5. New Challenges in Chemical Food Safety

Despite the comprehensive nature of the EU chemical food safety framework, several challenges persist. The adaptation of regulations to emerging risks—such as nanomaterials, microplastics, and new processing contaminants—is often slow due to the need for extensive scientific evaluation. Additionally, analytical limitations make it difficult to detect and quantify contaminants at very low concentrations, posing challenges in setting enforceable safety limits. The globalization of the food trade and new technologies add another layer of complexity, representing both a challenge and an opportunity to implement the safety of foods throughout the supply chain. Moreover, differences in national implementation of EU regulations can lead to discrepancies in enforcement, necessitating greater harmonization efforts [138].

### 5.1. Emerging Contaminants

Emerging contaminants (ECs) are artificial or naturally occurring chemicals increasingly detected in landfill leachate. They might have serious implications for human health and the environment. The reservoir of ECs is municipal solid waste, with production of around 2 billion tonnes of waste annually. Landfill leachate constitutes a critical source of ECs from the gradual breakdown of materials, in combination with rainwater and surface water seepage [139]. Major threats to both human health and the ecological balance of the environment arise from ECs [140]. People and natural ecosystems are being affected by the release of the following substances into the environment such as PPCPs (pharmaceutical and personal care products), PFASs, pesticides, industrial chemicals, cyanotoxins, nanomaterials, micro/nano-plastics, and other exogenous substances. Developed synthetic substances arise from mixing with other pollutants or are released into the global natural environment as breakdown products, and these can be dangerous at minute doses [2,141].

#### 5.1.1. PFASs

These are anthropogenic organic chemicals, potentially reaching 7 million chemicals [142,143]. They are persistent, bioaccumulative, and toxic (PBT) and are fluorinated pollutants [144]. The major sources of poly- and perfluorinated alkyl substances (PFASs) are landfills, aqueous film-forming foams in firefighting training, along with industrial and municipal sewage effluents [145]. The presence of PFASs is everywhere in the air, water, and biota globally. Ocean currents and the atmosphere facilitate the long-range transport of PFASs and their arrival in remote regions [146]. Their thermal stability and environmental persistence cause their transportation to surrounding water bodies, thus leading to increased contamination by PFASs in surface water. PFASs such as PFOA (perfluorooctanoic acid) and PFOS (perfluoro-octane sulfonate) do not biodegrade or hydrolyze. PFAS bioaccumulation in fish liver and muscle and food webs biomagnification is evident [147]. The fish protein-rich tissues are the home of PFASs, whereas other well-known POPs, like dioxins, reside in fatty tissue. The increase in carbon chain length increases the bioaccumulation potential of PFASs, as reported by Giesy et al. [148].

The toxicity of PFASs is caused by lowering immune function, increasing thyroid dysfunction, leading to liver- and kidney diseases and creating reproductive dysfunctions; increased cholesterol levels; and developmental, neurological, cancer, and immunological disorders [149]. Therefore, regulation of several PFASs nationally and/or internationally through the Stockholm Convention was carried out, and their use was banned [150].

The quantification and detection of PFASs can be complex due to the physical, chemical, and biological transformations of PFASs in marine environments, such as photochemical, biodegradation, particulate adsorption, and bioaccumulation. The morphometric and oxidative stress biomarkers as biomonitoring tools were utilized to evaluate the physiological and ecological impacts of PFAS exposure to marine organisms in coastal and freshwater environments [151,152,153,154].

PFASs were detected in various food matrices, including fish, meat, dairy products, eggs, and drinking water. Among PFASs, compounds such as PFOA and PFOS were the most studied and are associated with adverse health effects, including immunotoxicity, developmental toxicity, and potential carcinogenicity. In response to growing evidence, the EFSA established a tolerable weekly intake (TWI) for a group of four PFASs (PFOA, PFOS, PFNA, and PFHxS) in 2020, set at 4.4 ng/kg body weight/week. Regulatory measures, including the restriction of PFAS use and monitoring programs in food and water, have been introduced at the EU level to reduce exposure and protect public health [155]. As a consequence, the EU established MLs for this group of PFASs in certain foods and introduced them in 2022 in contaminant regulation [54].

#### 5.1.2. Microplastics and Nanoplastics

Microplastics (MPs) and nanoplastics (NPs) are primarily originating from the degradation of larger plastic debris. Their detection in seafood, table salt, bottled water, and other food products raises concerns about potential health risks associated with chronic exposure. MPs are classified into two main types: primary and secondary. They represent small plastic particles measuring less than 5 mm. Small sizes are characteristic of primary MPs, whereas the origin of secondary MPs is the breakdown of larger plastic objects (bottles, bags, and fishing nets) through environmental processes, including heat, ultraviolet radiation, and mechanical forces [156]. MPs have an increased surface-area-to-volume ratio, which enhances their chemical reactivity and potential for environmental dispersal and transportation due to their reduced size [157]. Their highly hydrophobic nature, along with their small size, enhances the absorption of these plastic fragments by living organisms and, hence, their binding with other harmful compounds [156]. Similarly, NPs—plastic particles smaller than 100 nm—also gain attention due to health concerns. These particles exhibit increased reactivity due to their even smaller size [158]. Polyethylene (PE), polypropylene (PP), and polystyrene (PS), along with polyethylene terephthalate (PET), polyvinyl chloride (PVC), and polymethyl methacrylate (PMMA), constitute some of the most commonly identified polymers.

Vectors for harmful pollutants, including organic chemicals, additives, biological agents, and TTEs, can be MPs and NPs [159,160]. They are detected in semen, feces, breastmilk, blood, thrombi, colon, atheroma, and liver [161,162,163,164,165].

Recent studies link MP consumption to several diseases causing multisystemic damage, affecting different systems such as the digestive, cardiovascular, neurological, and reproductive systems. They have been associated with inflammatory bowel disease, colorectal cancer, gut barrier dysfunction, non-alcoholic fatty liver disease, and cardiovascular aging [166,167,168,169].

Data show the intake by humans of up to 5 g/week of MPs through multiple exposure routes [170].

The EFSA began assessing the risks of MPs and NPs in 2016. While EFSA acknowledged the presence of MPs in food—especially in marine organisms such as mussels and fish—at that time, it concluded that there was insufficient data to conduct a full RA, particularly due to limited information on absorption, distribution, metabolism, excretion (ADME), and toxicity of plastic particles, especially at the nanoscale. Several analytical, methodological, and occurrence data gaps and uncertainties were also underlined. The EFSA also called for more research on MPs and NPs bioavailability and long-term health impacts [171,172]. Although no tolerable intake levels or legal limits have yet been established in the EU, EFSA continues to monitor developments, supporting further research to fill critical data gaps and guide future regulatory action.

#### 5.1.3. Novel Maillard Reaction-Derived Chemical Contaminants

**Figure 4:**
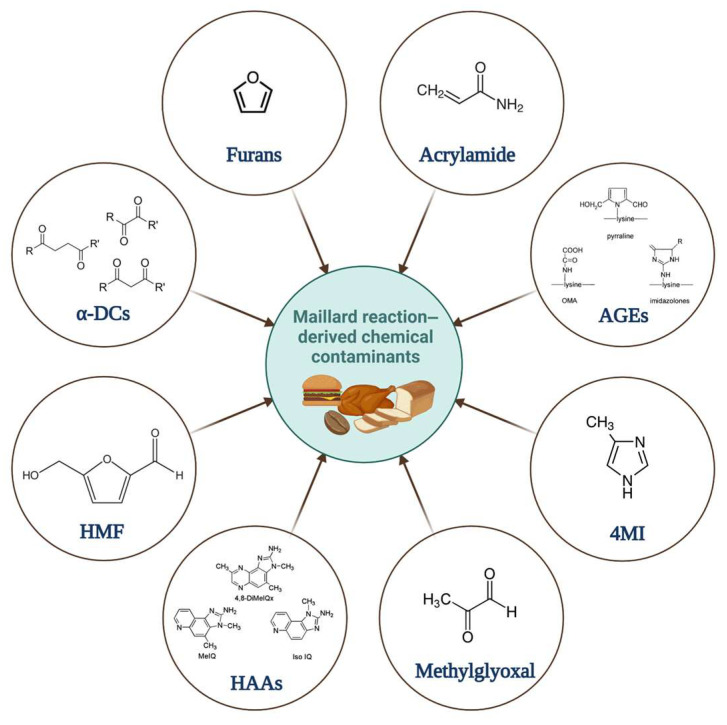
Maillard reaction-derived chemical contaminants.

Maillard reaction-derived chemical contaminants, such as acrylamide, heterocyclic aromatic amines (HAAs), advanced glycation end products (AGEs), 5-hydroxymethylfurfural (HMF), 4-methylimidazole (4-MI), methylglyoxal (MGO) and α-dicarbonyl compounds (α-DCs) are toxic chemicals produced during the thermal processing of certain foods (Figure 4). Although many of them are well-known toxicants, others are new substances of high concern for which no specific regulatory interventions or monitoring plans have been established yet [173].

Associations between the consumption of thermally processed foods and the incidence of diabetes, hypertension, cardiovascular and cerebrovascular disorders, cancer, and obesity have been reported [174,175,176]. Moreover, chemical contaminants produced by the Maillard reaction-derived chemical contaminants are genotoxic, mutagenic, and carcinogenic. Acrylamide and 2-amino-3-methyl-3H-imidazo [4,5-f]quinoline (IQ) were categorized by the IARC as class 2A carcinogens and furan, 4-MI, and others as class 2B carcinogens, respectively [177,178,179,180,181,182,183].

Extensive research on the presence of acrylamide in food since 2015 was published by EFSA, providing scientific advice to support EU-wide efforts and reduce the exposure of such products to consumers. The health implications of acrylamide and suggested mitigation strategies for the formation of acrylamide in food products in 2005 and 2010 were reviewed by the Joint FAO/WHO Expert Committee on Food Additives [72,73,184].

HMF is found in honey, syrups, fruit juice, fruit concentrates, baked goods, confectionery, and coffee. AGEs are present in grilled, roasted, and fried meat and heat-treated dairy products. Furan is usually formed in canned and jarred foods, roasted coffee, and baked goods [185,186,187].

### 5.2. Artificial Intelligence

Artificial intelligence (AI), as an emerging and important technology, has gradually gained attention. AI may replace some human processes of learning, reasoning, and problem-solving while also possessing perceptual, understanding, and creative abilities. Food processing, food quality inspection, food safety RA and analysis, and nutritional balance formulation could be affected by AI [188,189]. It is envisaged that in the near future, we can create an advanced, safe, and reliable AI-driven industrial chain that meets the needs of consumers.

Identifying food types and analyzing nutritional components are essential steps for AI in analyzing food properties, designing meal plans, and promoting comprehensive and balanced nutritional intake for all population groups. Kim et al. used two AI methods to authenticate infant food packaging. They first recognized certification marks using object detection to obtain the certification status of the infant food group for the collected front-of-pack (FOP) images. Moreover, they used optical character recognition to automatically extract nutrition and health-related texts from the images [190]. Convolutional Neural Networks (CNN) also have the ability to identify food characteristics. They constitute a deep learning model to process grid-structured data, such as images and videos. By extracting and analyzing local features, CNN can recognize different foods. Nfor et al. proposed a food recognition model based on CNN and Vision Transformer [191]. The hybrid model utilizes CNN ability to capture local features, such as edges and textures, incorporating model global dependencies and contextual relationships across the entire image. This helps with food recognition and retrieval of nutritional information.

AI provides intelligent and automated solutions for maintaining food safety. AI can automate quality control to identify and mark foreign objects and contaminants in food, thereby ensuring food safety [192,193,194]. Hyperspectral imaging, often combined with AI, has been used to detect toxins in food and has been used in food processing for many years now [159]. Mohammadpour et al. found that the use of the GNB algorithm could better assess the health risks of nitrites in toddlers’ and children’s food, thereby ensuring food safety [195]. AI can monitor and detect food physicochemical properties and analyze potential quality changes. Rivas et al. constructed a reduced order model (ROM) to monitor conditions such as temperature, oxygen, and water concentration during the refrigeration of fruits, hence automatically controlling changes during storage. They used the proper orthogonal decomposition method [196]. Similarly, the reduced-order modeling procedure of TwinLab was described by Kannapinn et al. [197]. Since its inception a decade ago by Grieves and Vickers, substantial efforts have been carried out to shape and standardize the digital twin concept, also establishing it as a dedicated research domain [198,199].

### 5.3. Multi-Source Data Fusion

Multi-source data fusion (MSDF) has emerged as a promising solution for comprehensive food safety analysis through the integration of multiple analytical techniques [200]. MSDF is a robust interdisciplinary approach synergistically integrating data from multiple sources, including different sensors and various data types from the same sensor, to enable comprehensive and accurate food safety evaluation [201,202]. This includes the integration of key analytical techniques, including spectroscopic methods (near-infrared, mid-infrared, Raman), chromatographic analysis, hyperspectral imaging, electronic noses, and chemical analyses. Fusion architectures and levels, preprocessing requirements, and advanced data analysis techniques, including machine learning and chemometrics, are described [203]. By extracting both redundant and complementary information, MSDF enables improved reliability in predicting safety attributes that depend on complex interactions among multiple factors [204,205]. The analytical determination of chemicals enforced with new chemometrics enhanced tools showed their potential for the development of innovative, refined, and eco-friendly multi-analyte and multi-class methods (e.g., mycotoxins, masked toxins, multi-element analysis) in complex food matrices [206,207,208].

## 6. Conclusions and Future Directions

Maintaining chemical food safety within the EU is a continuously evolving challenge, requiring a flexible and science-driven regulatory approach. Although existing regulations and sector-specific rules provide a robust foundation, future advancements must address emerging policies and goals. The EU is actively working to strengthen food safety through integrated strategies that prioritize public health, environmental protection, and technological innovation. A major step forward is compliance with the One Health approach, which aims to balance and optimize the health of humans, animals, and environment, also in food safety. In this way, new policies that prevent contamination at the source have been established, ensuring that chemical safety is addressed not only at the consumer level but throughout the entire food production and distribution chain. Therefore, EFSA is actively collaborating with other authorities, including the European Chemicals Agency (ECHA) and the European Medicines Agency (EMA), in several initiatives such as “One substance, one assessment”, which harmonizes chemical evaluations across different regulatory agencies, reducing redundancy and enhancing efficiency. Moreover, several new key policy initiatives are shaping the future of chemical food safety in the EU. As an example, the End-of-Waste Directive initiative promotes the safe reuse and recycling of materials in food production. While circular-economy strategies offer sustainability benefits, they also introduce new risks associated with chemical contaminants, requiring the development of advanced analytical techniques for RA. This, along with other initiatives, are enforcing the European Green Deal (EGD) and its derivatives, such as the Farm to Fork Strategy, Biodiversity Strategy and REFIT, transforming the EU food systems to be fair, healthy, and environmentally friendly. In the context of chemical safety, this strategy calls for stricter controls on contaminants, pesticide residues, and veterinary drugs while promoting alternative, sustainable agricultural practices. Another fundamental introduction to the EGD, which has a direct impact on chemical food safety, is the Chemicals Strategy for Sustainability (CSS). This initiative focuses on minimizing hazardous chemical exposure while advancing innovation in safer and more sustainable chemicals.

Finally, the improvement of accuracy and efficiency of chemical safety assessments is achieved through technological, analytical and methodological innovations. In this regard, the EU is actively incorporating NAMs into its regulatory framework. NAMs, both as stand-alone and integrated strategies, encompass advanced computational modeling, *in vitro* testing, and high throughput screening techniques, reducing reliance on traditional animal studies while enhancing predictive toxicology capabilities. In addition, innovations in high-resolution mass spectrometry, multi-omics data integration, machine learning, and bioinformatics are revolutionizing how chemicals are detected and quantified. These technological advancements will play a crucial role in addressing the new challenges in food safety.

In summary, the future of chemical food safety in the EU depends on the successful integration of regulatory and research needs, policy, and sustainability. Collaboration among regulatory bodies, scientific institutions, and industry stakeholders will be crucial to maintaining strong standards while promoting long-term improvements in food system practices.

## Figures and Tables

**Table 2:** Chemicals in food classified by key laws.

Chemical Class	Subclasses	Key Regulations	Notes	Ref
Chemical Contaminants	Mycotoxins (aflatoxins, ochratoxin A, patulin, deoxynivalenol, zearalenone, fumonisins, citrinin, ergot sclerotia, and ergot alkaloids) Plant toxins (erucic acid, tropane alkaloids, hydrocyanic acid, pyrrolizidine alkaloids, opium alkaloids, Δ9-THC) Metals and other elements (lead, cadmium, mercury, arsenic, inorganic tin) PCBs and Dioxins Perfluoroalkyl substances Processing contaminants (polycyclic aromatic hydrocarbons (PAH): benzo(a)pyrene, sum of 4 PAHs; 3-monochloropropane-1,2-diol (3-MCPD), glycidyl fatty acid esters) Others (nitrates, melamine, perchlorate)	Regulation (EU) No. 2023/915	Establishes maximum levels for contaminants in food	[54]
Marine Biotoxins	paralytic shellfish poison (PSP), amnesic shellfish poison (ASP), okadaic acid and dinophysistoxins, yessotoxins, azaspiracids	Regulation (EC) No. 627/2019	Establish maximum levels and control plans	[81]
Acrylamide		Regulation (EU) No. 2017/2158	Implementation of acrylamide reduction measures	[82]
		Recommendation (EU) No. 2019/1888	Monitoring the presence of acrylamide in certain foods	[74]
*Alternaria* Toxins	alternariol, alternariol monomethyl ether, and tenuazonic acid	Recommendation (EU) No. 2022/553	Monitoring the presence of *Alternaria* toxins in food	[75]
Food Additives	26 functional classes (sweeteners, colors, preservatives, antioxidants, carriers, acids, acidity regulators, anti-caking, anti-foaming and bulking agents, emulsifiers, emulsifying salts, flavor enhancers, firming, gelling, glazing, raising and foaming agents, humectants, modified starches, packaging gases, propellants, sequestrants, stabilizers, thickeners, flour treatment agents)	Regulation (EC) No. 1333/2008	Defines approved food additives, their conditions of use, and maximum levels	[11]
		Regulation (EU) No. 231/2012	Purity criteria of food additives	[83]
Flavorings	flavoring substances, flavoring preparations, thermal process flavorings, smoke flavorings, flavor precursors, or other flavorings or mixtures	Regulation (EC) No. 1334/2008		[40]
Pesticide Residues	acaricides, bactericides, fungicides, herbicides, insecticides, larvicides, rodenticides	Regulation (EC) No. 396/2005;	Sets MRLs for pesticides	[41]
		Regulation (EC) No. 1107/2009	placing of plant protection products on the market	[45]
		Directive No. 2009/128/EC	promotes sustainable pesticide use	[12]
Veterinary Drug Residues	antibiotics, hormones, anabolic steroids, FANS (…)	Regulation (EU) No. 37/2010	Establishes MRLs for veterinary medicinal products in food-producing animals.	[47]
		Regulation (EC) No. 470/2009	Outlines the process for determining MRLs for veterinary medicinal products in food.	[48]
		Regulation (EU) No. 2019/1871	Establishes reference limits for unauthorized pharmacologically active substances detected in food of animal origin	[84]
		Regulation (EU) No. 2019/6	Specifies the rules governing the approval and use of veterinary medicinal products	[85]
Food Contact Materials	monomers, other starting substances, macromolecules obtained from microbial fermentation, additives, and polymer production aids contaminants	Regulation (EC) No. 1935/2004	Establishes safety requirements and migration limits for materials in contact with food.	[50]
		Regulation (EU) No. 10/2011	Criteria and authorization of plastic materials and articles intended to come into contact with food	[49]

**Table 3:** Insights from pairwise assessments of *in vivo* genotoxicity BMD estimates across multiple endpoints.

Substance	Study Model	Endpoint	Tissue	BMDL	Ref
Benzo[a]pyrene	Mouse	GM	liver and small intestine	8.47 and 0.75, respectively	[109]
Ethyl methanesulfonate	Mouse	GM	liver and spleen	2.3 and 0.35, respectively	[110]
Ethyl methanesulfonate	Mouse	GM	liver and bone marrow	41 and 9.3, respectively	[111]
1-Methyl-1-nitrosourea	Rat	GM	peripheral blood	0.2	[112]
Aristolochic acids	Rat	GM	peripheral blood	5.3	[113]
Ethylene oxide	Mouse	CA	peripheral blood	20.4	[114]
Temozolomide	Rat	MN	peripheral blood and liver	0.3 and 2.5, respectively	[115]

GM—gene mutation; CA—chromosomal aberrations; MN—micronucleus formation.

**Table 4:** Validation of analytical methods for the determination of chemicals in foods: parameters and general requirements [81,132,134,135].

Parameter	Description	Main Acceptance Criteria
Selectivity/Specificity	The ability of the method to distinguish the analyte from the possible interferences	No interferences near the analyte signal (e.g., ±5% retention time in chromatographic methods)
Limit of Detection (LOD)	The minimum reliably detectable amount of an analyte	Method-specific LOD/LOQ thresholds
Limit of Quantification (LOQ)	The lowest concentration that can be reliably quantified	Method-specific LOD/LOQ thresholds
Linearity	The ability to obtain test results that are directly proportional to the concentration of the analyte in the sample	R² > 0.98–0.99
Accuracy	The closeness of an analytical measurement to the true or accepted reference value; it is described in ISO 5725-1 as the sum of precision and trueness	It is described in ISO 5725-1 as the sum of precision and trueness
Precision	The closeness of agreement between the measured values obtained by the replicate measurements on the same or similar objects under specified conditions; generally estimated as (relative) standard deviation (RSD) or coefficient of variation (CV). Precision can vary depending on the level of variability considered: (1) repeatability refers to closeness of results when the same sample is measured under the same conditions within a short period (usually one day); (2) intermediate precision involves precision over a longer period in a single lab, accounting for more variables like different analysts or reagents; (3) reproducibility refers to precision of results across different laboratories (important when methods are standardized or used in multiple labs).	Intermediate precision (n ≥ 6) CV(%) < 5–25 RSD < 15%
Trueness	The agreement between a reasonably large number of measurements and true value (reference value), generally estimated as recovery (R)	R(%) = 70–120
Robustness	Stability of method performance under varying conditions	*minor changes* (e.g., pH, mobile phases) *major changes* (matrix)
Matrix effect	An influence of one or more co-extracted compounds from the sample on the measurement of the analyte concentration or mass. It may be observed as an increased or decreased detector response compared with that produced by solvent solutions of the analyte	ME(%) ≤ 20
Uncertainty	A range around the reported result within which the true value is expected to fall with a specified level of confidence, typically 95%	U ≤ 50% of MRL for contaminants
